# Lifespan of companion dogs seen in three independent primary care veterinary clinics in the United States

**DOI:** 10.1186/s40575-020-00086-8

**Published:** 2020-06-16

**Authors:** Silvan R. Urfer, Matt Kaeberlein, Daniel E. L. Promislow, Kate E. Creevy

**Affiliations:** 1 Dog Aging Project, Department of Pathology, University of Washington School of Medicine, Seattle, WA USA; 2 Department of Biology, University of Washington, Seattle, WA USA; 3 College of Veterinary Medicine & Biomedical Sciences, Texas A&M University, College Station, TX USA

**Keywords:** Dogs, Veterinary medicine, Epidemiology, Epizootiology, Survival analysis, Life span, Lifespan, Healthspan, Inbreeding, *One health*, *One welfare*

## Abstract

**Background:**

The privately owned companion dog is an emerging model in comparative medicine, notably because it shares the human environment including its risk factors, is affected by many analogous age-related diseases, receives comparable medical care, and has excellent veterinary medical data available.

Past studies of dog lifespan have used academic, corporate or insurance data. While independent primary care data exist for the UK, none have as of yet been published for the US. This study analyzed data from three independent primary care US veterinary hospitals and identified factors that influence lifespan and mortality in a cohort of *n* = 20,970 privately owned dogs using Kaplan-Meier survival estimators and Cox Proportional Hazards modelling, including body size as a covariate.

**Results:**

As previously reported, body size was negatively correlated with lifespan. Gonadectomy was associated with a longer lifespan, with the effect being stronger in females than in males. This lifespan advantage was conserved in gonadectomized female dogs that lived to at least ages 5 and 8 years. We did not find significant differences in lifespan between purebred and mixed breed dogs; however, breeds with larger effective population sizes and/or lower inbreeding coefficients had median survival times 3–6 months longer than breeds with smaller effective population sizes or higher inbreeding coefficients, indicating that these measures of genetic diversity may be affecting breed lifespans. We also found that dog breeds belonging to the “Mountain” ancestral group had median survival times that were 3.5–4.6 years shorter than other purebred dog groups, which remained significant even when correcting for body size.

**Conclusions:**

Our findings show that it is possible to obtain and analyze data from independent veterinary clinics in the US, an approach that could be useful for studies of comparative epidemiology under the *One Health* and *One Welfare* paradigms. We also show that the lifespan effects of gonadectomy are not identical between the sexes and should be investigated separately by sex in future analyses. More research is needed to further clarify the influence of age at gonadectomy, as well as the factors leading to the observed differences in lifespan in the “Mountain” ancestral group and in dog breeds of varying inbreeding coefficients and effective population sizes.

## Plain English summary

Studying health and lifespan in dogs is important both for the dogs themselves and also because it is important for human health and well-being. Dogs live in the same environment as humans, receive similar care, and get many of the same diseases. The Dog Aging Project (www.dogagingproject.org) uses veterinary data from dogs to benefit both dog and human health.

Several types of data have been used to study lifespan in dogs, including university hospital records, corporate practice records, and pet insurance data. Private practice data exist for the UK, but have not been published for the US. We collected data from three private US veterinary clinics over 1 year, representing 20,970 dogs, of which 1535 (7.3%) died during this year. We had information on age, breed, weight, sex, gonadectomy status, and cause of death if applicable, and then studied how these factors influence lifespan in dogs.

We found that large dogs do not live as long as small dogs on average, which is known and increases our confidence in our data. We also found that dogs that were gonadectomized lived longer than intact dogs, with a bigger effect in females than in males. While we did not find a lifespan difference between purebred and mixed breed dogs, we found that dog breeds with more genetic diversity lived longer than breeds with less, as measured by inbreeding coefficients and effective population sizes. Additionally, dogs in a specific genetic cluster of breeds (“Mountain ancestral group”) tended to live shorter, and this difference is not explained by their larger size.

This is the first large study of lifespan in dogs seen in non-corporate private veterinary practice in the US. We are able to collect and analyze such data, which will be useful as the Dog Aging Project progresses. We also show that gonadectomy appears to have different effects in males versus females and needs to be considered separately by sex, and that breeds with more genetic diversity appear to live longer than those with less genetic diversity. More research is needed to determine whether age at the time of gonadectomy impacts survival, and to understand what causes the decreased lifespan in the Mountain ancestral group breeds.

## Background

The privately owned companion dog (*Canis lupus familiaris*) is an emerging model in the field of geroscience, notably because it shares the human environment including its risk factors, is affected by many of the same age-related diseases, receives comparable medical treatment, and has excellent veterinary data available [1]. The concept that a shared environment affects health outcomes across species in similar ways is referred to as *One Health* and has interesting implications for the use of non-human animals as models and sentinel organisms for human epidemiology [2]. Aging is the single most important risk factor for a wide variety of otherwise unrelated diseases in humans, and better comprehension of natural aging can therefore be expected to improve our understanding of the development of diseases such as cancer, heart disease, type 2 diabetes, various forms of dementia, and kidney failure [3]. In this context, the Dog Aging Project (www.dogagingproject.org) endeavors to establish the companion dog as a model not only for aging in dogs, but also for human aging, using both descriptive and interventional approaches [4–6].

Two main approaches have been commonly used in veterinary epidemiological studies in large US dog populations:

1. Studies based on the veterinary medical database (VMDB), which compiles data collected from North American veterinary teaching hospitals, as well as studies that have used other databases derived from veterinary teaching hospital records [7–12].
2. Studies based on data from a single corporate primary care practice chain that uses a consistent medical record system in all of its clinics [13–18].

Two additional methods have been used in studies of large European dog populations:

1. Aggregation of data from primary care practices using diverse medical records systems (VetCompass) [13, 16, 17].
2. Studies based on data from pet insurance companies, which are taken from records of claims from large pools of insured animals (e.g., [19, 20]).

Each one of these approaches entails its own advantages and challenges. Cases seen at veterinary teaching hospitals exhibit referral bias [21] and thus do not accurately represent the average lifespan or most common diagnoses of the entire US dog population. While the large corporate practice database is fully standardized as far as diagnoses are concerned, its use is limited by the fact that the data are proprietary, which means that the raw data cannot be made publicly available. Also, as a single nationwide practice, these clinics exist in neighborhoods of relatively similar socioeconomical and suburban makeup across the country and serve a particular clientele, which again may make their data less generalizable to the total US dog population [18]. The UK VetCompass system represents an example of data accumulated from diverse, non-affiliated private practices [13, 16, 17, 22, 23]; however, UK dogs likely differ from US dogs in demographics, as is reflected in the differences in breed popularity rankings between the American Kennel Club (AKC) [24] and the UK Kennel Club (KC) [25]. As such, UK dogs may also differ from US dogs in individual disease risks. Finally, studies based on insurance data usually classify diagnoses in a standardized fashion; however, some companies cease to offer coverage after the animal reaches a certain age, providing an incomplete picture of lifespan and causes of death in the population. Furthermore, in the US, less than 5% of owners choose to purchase companion animal insurance [26], meaning that the use of insurance data to study US dogs would be biased by the characteristics of the small subset of US dog owners who make this choice.

Dog lifespan has commonly been studied based on mortality data, in which information is limited to deceased animals and does not include dogs from the same birth cohorts that are still alive at the time the data are collected. This type of analysis causes data to be right-censored and will generally result in an underestimation of lifespan [27, 28]. If data from dogs that are still alive are available, there are methods that avoid this problem, including Kaplan-Meier analysis [29] and Cox proportional hazards regression [30], which take into account individuals known to have still been alive at a certain age even if we do not have information on their ages at death. In the case of Cox regression, we can analyze the effects on hazard of death of multiple variables simultaneously. When using these methods, median survival time (MST) can be compared among groups even before all individuals have a documented date of death. MST is likely a more clinically meaningful parameter than average lifespan calculated from only those individuals with known dates of death. Unfortunately, such methods have not been routinely applied to many of the existing studies on dog lifespan and causes of death thus far, which results in artificially low lifespan estimates and limits comparability between studies based on different populations with varying degrees of right censoring [27, 28].

These issues have resulted in a largely inconclusive body of evidence regarding factors that are associated with lifespan in dogs. It is generally agreed that lifespan in dogs is inversely correlated with body weight [12, 31, 32], even though considerable variation in life expectancy exists between different breeds within the same body size class [9, 18, 20, 33, 34]. It is also widely accepted that gonadectomized female dogs generally live longer than intact females of the same size [10, 17, 18, 20, 35]; however, the evidence is less clear regarding the effects of gonadectomy on lifespan in males [11, 35]. In addition, several studies have looked at the effects of gonadectomy without differentiating between sexes, which may result in the strong effect of female gonadectomy overwhelming the possible lack of such an effect in males, and making it appear that an overall effect exists [34, 35]. Furthermore, the influence of age at gonadectomy has not thus far been studied in any large population comprising multiple breeds. The fact that any gonadectomized dog needs to have lived to the age at which the procedure was carried out may also risk introducing some degree of bias [36]. In addition, small studies of the influence of gonadectomy on disease incidence within certain breeds suggest that age at the time of gonadectomy may affect incidence of certain diseases in certain breeds [37].

There is also a considerable body of research indicating that inbreeding coefficients are high and genetic diversity is low in many breeds [38–40]; however, direct correlations between inbreeding and/or genetic diversity and life expectancy in dogs have proven challenging to establish [41, 42], which may be at least partially mediated through the occurrence of purging in some breeds [43–45]. Additionally, “inbreeding”, “effective population size” and “genetic diversity” are not consistently defined across studies and may refer to data based on pedigree analysis conducted over differing numbers of generations, as well as data obtained through several different molecular methods, all of which raises issues of comparability between different studies.

In this study, we analyzed medical records data that had been collected from three private US veterinary hospitals in three different locations. Our goal was to describe demographics, survival curves, age at death, and causes of death among a diverse population of privately owned US companion dogs, and to demonstrate the feasibility of veterinary electronic medical record data collection from independent (non-corporate) veterinary practices using different medical records software. Breed and inbreeding status if purebred, weight, sex, and gonadectomy status were investigated for associations with age at death and cause of death.

## Results

**Fig. 1:**
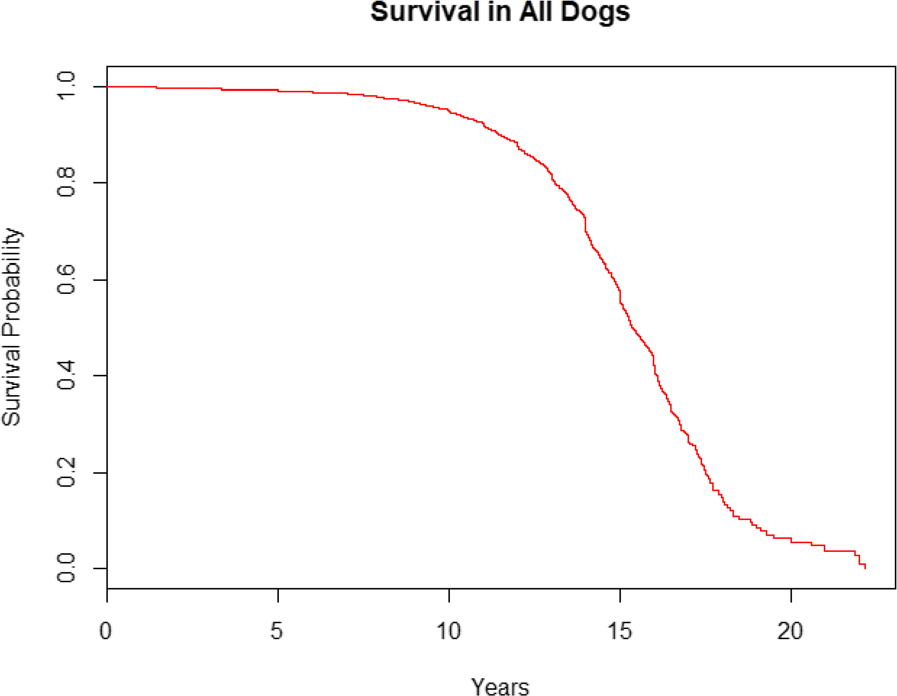
Kaplan-Meier survival curve for lifespan in all dogs (*n* = 20,970)

The data consisted of *n* = 32,179 data points, each of which represented one visit to one of the three clinics. Some of these data points were from dogs that visited their veterinarians more than once over the course of a year. After removing multiple entries from the same patients, *n* = 20,970 individual dogs remained, of which *n* = 1535 died during the course of the year, representing 7.3% mortality over a one-year period. The overall Kaplan-Meier survival curve for lifespan in the whole population is provided in Fig. 1.

Of the *n* = 20,970 dogs in the data, 13,068 (62.3%) were classified as purebreds, 6480 (30.9%) were mixed breeds, and 1085 (5.2%) were F1 hybrids. Purebred/mixed breed/hybrid status was unknown in 337 dogs. A list of the 10 most common breeds and their percentage relative to all purebreds is provided in Table 1. A list of the F1 hybrids and their percentage relative to all F1 hybrids is provided in Supplemental Table 1. The number of dogs per AKC and ancestral group is provided in Table 2.

**Table 1:** List of the 10 most common breeds in the data, their percentage in relation to all purebreds (*n* = 13,086), and median survival times (MST) in years with 95% confidence intervals

Breed	N	%	MST (95% CI)
Labrador Retriever	1419	10.9%	14.3 (14.0–14.9)
Golden Retriever	767	5.9%	14.0 (13.6–16.7)
Greyhound	577	4.4%	14.3 (13.9-NA)
Yorkshire Terrier	497	3.8%	18.0 (17.2-NA)
Chihuahua	484	3.7%	16.0 (14.1-NA)
Shih Tzu	442	3.4%	16.5 (16.2-NA)
German Shepherd	376	2.9%	13.4 (13.0-NA)
Beagle	374	2.9%	16.1 (16.0-NA)
Dachshund	317	2.4%	16.3 (16.0-NA)
Boxer	287	2.2%	13.2 (11.8-NA)

**Table 2:** List of purebreds by AKC group and Ancestral group, including Median Survival Time (MST) in years and 95% Confidence Intervals

**AKC Group**	**N**	**%**	**MST (95% CI)**
Herding	1076	8.8%	15.2 (14.8–16.1)
Hound	1704	14.0%	15.6 (15.2–16.2)
Non-Sporting	1394	11.4%	15.4 (15.2–16.1)
Sporting	3067	25.2%	14.6 (14.2–14.9)
Terrier	937	7.7%	15.8 (15.4-NA)
Toy	2795	23.0%	16.2 (15.9–16.7)
Working	1205	9.9%	14.2 (13.0–16.1)
**Ancestral Group**	**N**	**%**	
Ancient	255	2.5%	15.6 (14.2-NA)
Herding-Sighthound	1197	11.8%	14.9 (14.3–15.7)
Mastiff-Terrier	4288	42.3%	15.1 (14.9–15.9)
Modern	4143	40.9%	16.0 (15.6–16.2)
Mountain	258	2.5%	11.4 (11.1-NA)

1551 (7.4%) of the dogs were intact females, 2115 (10.1%) were intact males, 8711 (41.5%) were gonadectomized females and 8567 (40.9%) were gonadectomized males. Twenty-six dogs were of unknown sex. According to measured weight class, 5061 (28.3%) of the dogs were small, 5248 (29.4%) were medium, 6179 (34.6%) were large and 1097 (6.1%) were giant-sized. We did not have measured weights for 267 dogs, and 3118 dogs were not included because they were under 18 months old. By breed-specific weight class, 4197 (32.1%) of all purebreds were small, 3298 (25.2%) were medium, 4794 (36.7%) were large and 589 (4.5%) were giant-sized. Among purebred dogs, 190 could not be assigned to a breed-specific weight class due to ambiguous breed descriptions. By clinic, 2674 (12.8%) of dogs were from Clinic A, 14,671 (70.0%) were from Clinic B and 3625 (17.3%) were from Clinic C. The distribution of dogs by sex and body size as well as MST among clinics is provided in Supplemental Table 2.

Overall MST was 15.4 years (95% CI 15.2–15.7 years). MST in all females was 15.6 years (15.3–16.0 years); all males, 15.2 years (15.0–15.5 years); intact males, 15.0 years (14.0–15.5 years); gonadectomized males, 15.2 years (15.0–15.7 years); intact females, 14.1 years (13.5–15.5 years); gonadectomized females, 15.8 years (15.4–16.1 years). MST in purebreds was 15.5 years (15.2–15.8 years); mixed breeds, 15.2 years (15.0–15.9); and F1 hybrids, 16.1 years (15.2-NA).

**Fig. 2:**
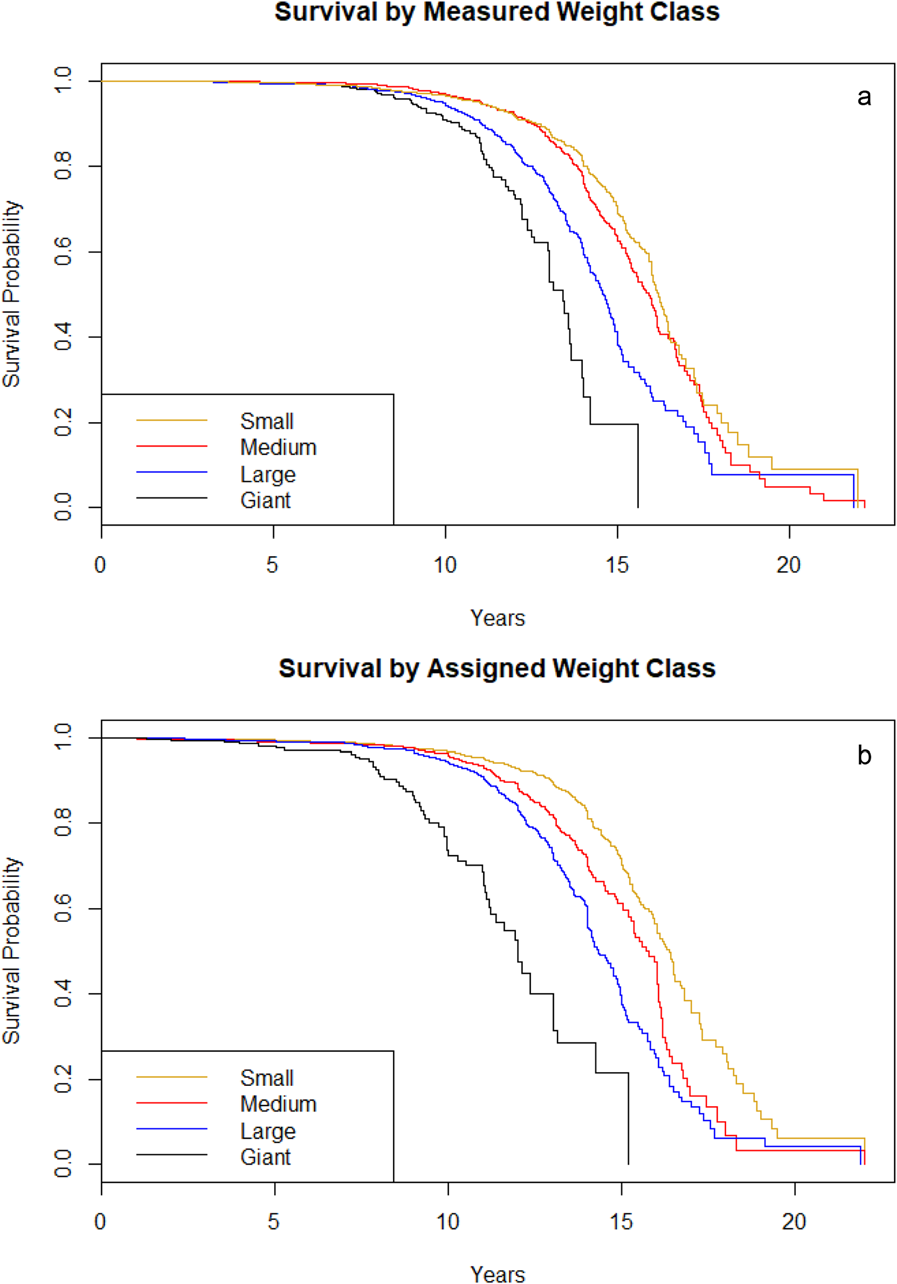
**a** and **b** Kaplan-Meier survival curves for measured weight in all dogs aged over 18 months, and all purebred dogs based on average breed weight as defined by the AKC

As expected, comparing dogs by the four body size categories using measured weight at age 18 months or over indicated significant differences in survival (χ^2^ = 209, *P* < 2E-16, d.f. = 3, log rank test). This was also the case when assigning purebred dogs to the four weight classes based on average weight as per the AKC breed standards (χ^2^ = 269, *P* < 2E-16, d.f. = 3, log rank test). MST according to measured weight class was 16.2 (16.0–16.5) years for small dogs, 15.9 (15.5–16.2) years for medium size dogs, 14.6 (14.4–14.9) years for large dogs, and 13.4 (13.0–14.0) for giant dogs. MST according to purebred weight class was 16.4 (16.0–16.7) years for small dogs, 15.7 (15.3–16.0) years for medium size dogs, 14.3 (14.1–14.8) years for large dogs, and 12.0 (11.2–13.0) years for giant dogs. Kaplan-Meier survival curves for both measured and assigned weight classes are provided in Fig. 2a and b.

**Fig. 3:**
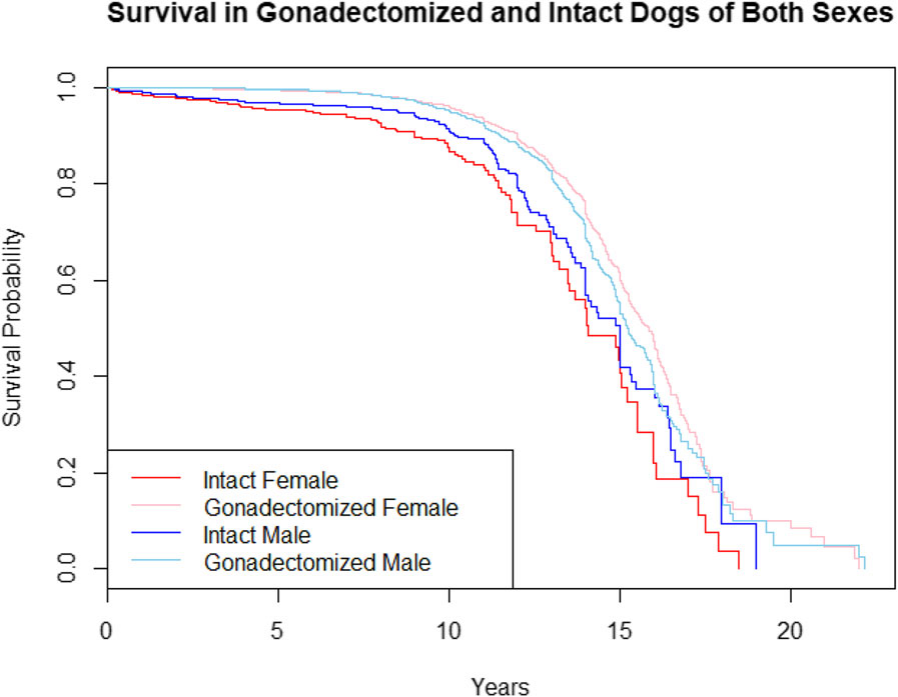
Kaplan-Meier survival curves for gonadectomized and intact dogs of both sexes

When comparing male and female survival curves regardless of gonadectomy status, female dogs lived significantly longer than male dogs (χ^2^ = 4.6, *P* = 0.032, d.f. = 1, log rank test); however, when correcting for weight and clinic, the effect on the hazard ratio (HR) was not significant (HR_male_ = 1.11; 95% CI = 1.00–1.24; *P* = 0.051, Cox proportional hazards model). When comparing survival within sexes by gonadectomy status, gonadectomized females lived significantly longer than intact females (χ^2^ = 106, *P* < 2E-16, d.f. = 1, log rank test). This effect remained significant when correcting for weight and clinic (HR_gonadectomized_ = 0.32, 95% CI = 0.26–0.41, *P* < 2E-16, Cox proportional hazards model). Gonadectomized males also lived significantly longer than intact males and this also remained significant when correcting for weight and clinic; however, the effect was weaker than that observed in females (χ^2^ = 32.7, *P* = 1E-8, d.f. = 1, log rank test; HR_gonadectomized_ = 0.62, 95% CI = 0.51–0.76, *P* = 3E-6, Cox proportional hazards model). Survival curves for all four categories are provided in Fig. 3.

Given the possibility that the observed survival advantage in gonadectomized dogs may be due to the fact that any gonadectomized dog needs to already have lived to the age of gonadectomy [36], we repeated these analyses using only dogs that were at least 5 years old. Doing this resulted in MST (95% CI) of 15.0 (14.1–16.0) years for intact males, 15.2 (15.0–15.7) for gonadectomized males, 14.9 (13.7–15.5) for intact females, and 15.9 (15.5–16.1) for gonadectomized females. In this analysis, a significant survival advantage of gonadectomized over intact females remained (χ^2^ = 34.5, *P* = 4E-9, d.f. = 1, log rank test), and this did not change when correcting for weight and clinic (HR_gonadectomized_ = 0.45, 95% CI = 0.34–0.60, *P* = 2E-8, Cox proportional hazards model). In contrast, while the survival advantage of gonadectomized over intact males remained significant in the direct comparison (χ^2^ = 5.8, *P* = 0.016, d.f. = 1, log rank test); it was not significant when correcting for weight and clinic (HR_gonadectomized_ = 0.82, 95% CI = 0.65–1.04, *P* = 0.097, Cox proportional hazards model).

Because it has been proposed that being intact may provide a survival advantage in female Rottweilers aged at least 8 years [46], we also tested the effect of gonadectomy in females known to have lived to be at least 8 years old. In doing so, we found an MST of 14.9 (14.0–15.5) years for intact and an MST of 15.9 (15.6–16.1) years for gonadectomized females. Statistically, the survival advantage of gonadectomized over intact females remained significant even under these constraints (χ^2^ = 27.5, *P* = 2E-7, d.f. = 1, log rank test), and also when correcting for weight and clinic (HR_gonadectomized_ = 0.45, 95% CI = 0.33–0.60, *P* = 1E-7, Cox proportional hazards model).

**Fig. 4:**
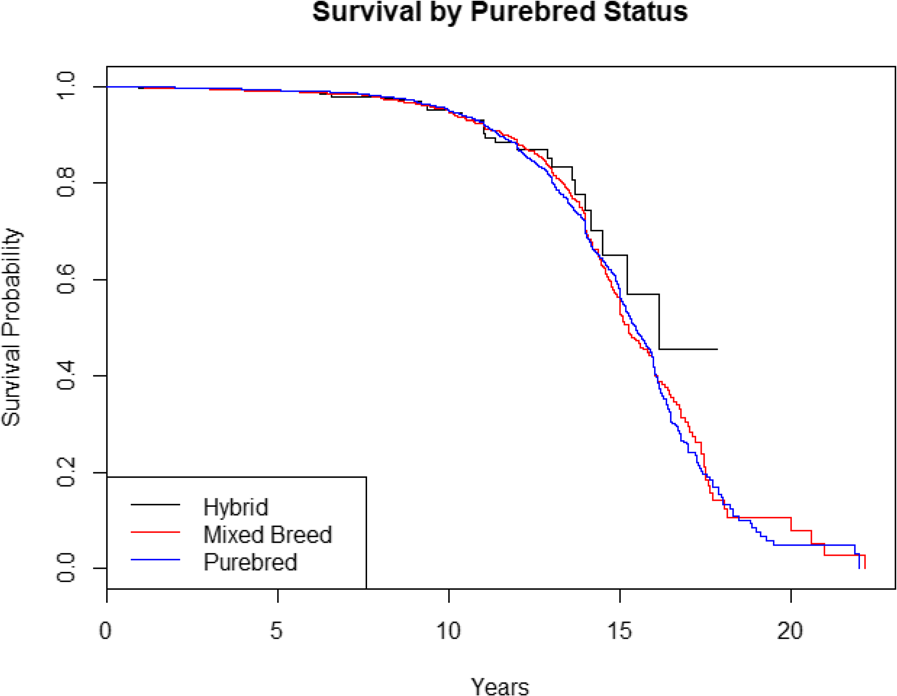
Kaplan-Meier survival curves for F1 hybrid, mixed breed, and purebred dogs

Comparing survival by purebred status in purebreds, mixed breeds and F1 hybrids indicated no significant differences (χ^2^ = 3.4, *P* = 0.183, d.f. = 2, log rank test). When only comparing purebreds and mixed breed dogs, this did not change (χ^2^ = 0, *P* = 0.945, d.f. = 1, log rank test). Similarly, when correcting for clinic and weight in a Cox model, no significant effects of purebred status on survival were found with or without F1 hybrids included. Kaplan-Meier survival curves of purebred, mixed breed and F1 hybrid dogs are provided in Fig. 4.

**Fig. 5:**
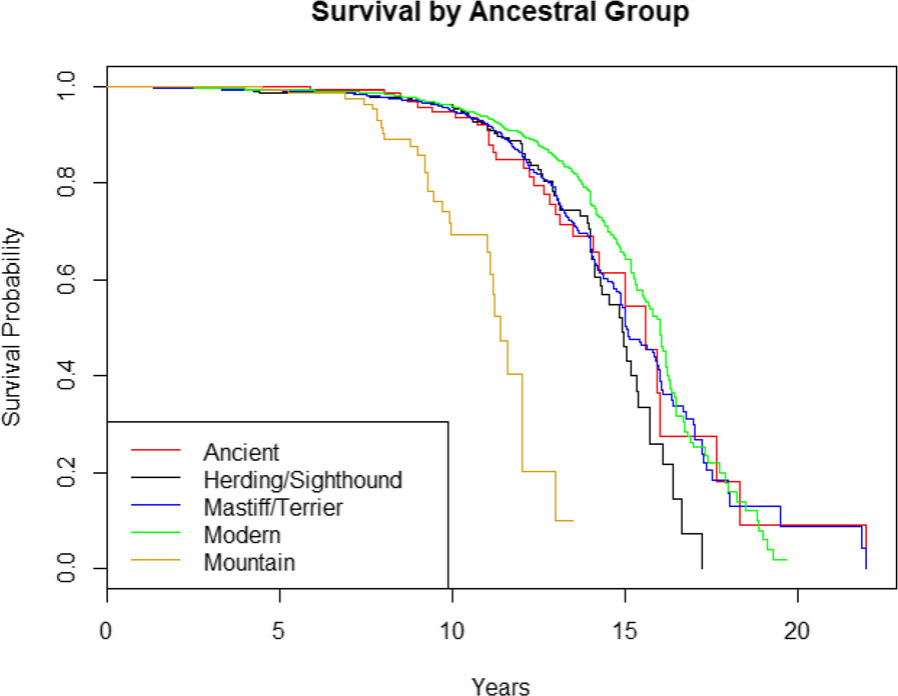
Kaplan-Meier survival curves by ancestral group

Dog breeds can be combined into seven historical and/or function-based groups as done by the American Kennel Club (AKC), or into five ancestral groups based on DNA analysis [47]. Comparing purebred dogs by AKC breed groups revealed significantly shorter lifespan for breeds belonging to the Working group even when correcting for weight and clinic (HR_Working_ = 1.57, 95% CI = 1.25–1.96, *P* = 7E-5, Cox proportional hazards model). Comparing purebred dogs by DNA-based ancestral group [47] revealed significantly shorter lifespan for dogs belonging to the Mountain group, which also remained significant when correcting for weight and clinic (HR_Mountain_ = 2.88, 95% CI = 1.89–4.40, *P* = 9E-7, Cox proportional hazards model). However, when combining both AKC and ancestral groups in the same model and correcting for weight and clinic, only the Mountain ancestral group lifespan remained significantly shorter (HR_Mountain_ = 3.14, 95% CI = 1.74–5.67, *P* = 0.0001, Cox proportional hazards model). Kaplan-Meier survival curves by ancestral group are provided in Fig. 5. MST and 95% confidence intervals for AKC and ancestral breed groups are provided in Table 2.

Using previously published breed-specific average inbreeding coefficients based on the purebred US dog population [48], we assigned purebred dogs to an above- and a below-median inbreeding category based on their breeds. For the breeds in our sample, median inbreeding was at a coefficient of inbreeding [49] of 0.263. Breeds with below-median inbreeding had a MST of 15.8 (15.3–16.0) years; those with above-median inbreeding had a MST of 15.3 (15.0–15.9) years. The statistical analysis showed significantly longer survival in dogs from breeds with below-median inbreeding coefficients compared to those with above-median inbreeding coefficients (χ^2^ = 12.7, *P* = 0.0004, d.f. = 1, log rank test). This remained true when using the coefficient of inbreeding for each breed as a continuous variable and correcting for weight and clinic (HR = 5.82, 95% CI = 2.73–12.38, *P* = 5E-6, Cox proportional hazards model).

**Fig. 6:**
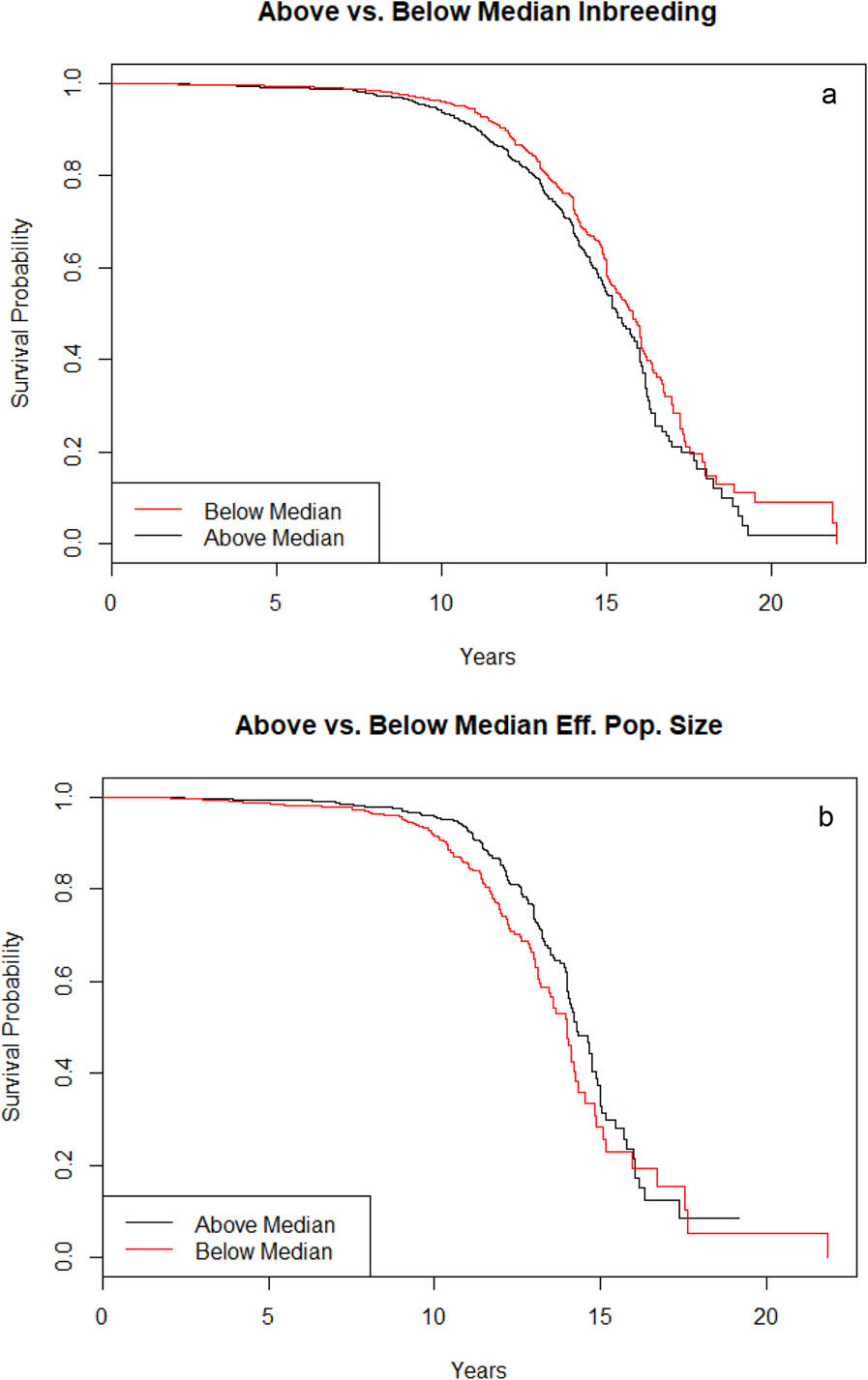
**a** and **b** Kaplan-Meier Survival Curves for above- vs. below median inbreeding coefficients and effective population sizes in purebred dogs

We also analyzed breed effective population size as previously defined [50] and assigned our purebred dogs to a below- and an above-median category based on their breeds, with the median effective population size being 67. This resulted in a MST of 14.3 (14.0–14.9) years for dogs belonging to breeds with above-median effective population size, and a MST of 14.0 (13.5–14.2) years in dogs belonging to breeds with below-median effective population size. This difference was statistically significant when comparing survival curves (χ^2^ = 10.6, *P* = 0.001, d.f. = 1, log rank test), and remained significant when correcting for weight and clinic (HR = 0.99, 95% CI = 0.99–1.0, *P* = 0.001, Cox proportional hazards model). Inbreeding- and population size-based survival curves are provided in Fig. 6a and b.

In the *n* = 1529 dogs for which death was reported during the year of this study, data on the organ system (OS, *n* = 950) and pathophysiologic process (PP, *n* = 800) causing death were also available. The most frequently identified OS cause of death was neurological (*n* = 200, 21.1%), and the most frequently identified PP cause of death was neoplasia (*n* = 364, 45.5%). The OS cause of death was unclassified for *n* = 585 (38.1%) dogs, and the PP cause of death was unclassified for *n* = 735 (47.9%) dogs.

Neoplasia was the most common pathophysiologic process causing death in all subcategories including purebreds, mixed breeds, males, females, sexually intact dogs, gonadectomized dogs, and all four size classes. The only exception to this were sexually intact female dogs, which were most likely to die from trauma. Large and giant-sized dogs were significantly more likely to die from neoplasia than small and medium-sized dogs (*P* = 0.0098, Fisher’s exact test). Causes of death by pathophysiologic process are provided in Table 3.

**Table 3:** Causes of death classified by pathophysiologic processes in our data

Pathophysiologic Process	All Dogs	%	Purebreds	%	Mixed Breeds	%	Males	%	Females	%	Intact	%	Gonadectomized	%	Small	%	Medium	%	Large	%	Giant	%	MI	%	MG	%	FI	%	FG	%
**cardiovascular**	14	1.75	8	1.60	5	1.90	7	1.76	7	1.75	0	0.00	14	2.13	4	2.92	3	2.68	0	0.00	1	2.86	0	0.00	7	2.19	0	0.00	7	2.06
**congenital**	12	1.50	9	1.80	2	0.76	9	2.26	3	0.75	6	4.29	6	0.91	3	2.19	1	0.89	5	2.43	0	0.00	4	5.06	5	1.57	2	3.28	1	0.29
**degenerative**	233	29.13	154	30.74	74	28.14	116	29.15	116	29.00	25	17.86	207	31.46	47	34.31	36	32.14	58	28.16	11	31.43	15	18.99	101	31.66	10	16.39	106	31.27
**infectious/inflammatory**	86	10.75	59	11.78	23	8.75	41	10.30	45	11.25	26	18.57	60	9.12	14	10.22	15	13.39	24	11.65	5	14.29	14	17.72	27	8.46	12	19.67	33	9.73
**metabolic**	21	2.63	13	2.59	7	2.66	13	3.27	8	2.00	4	2.86	17	2.58	6	4.38	3	2.68	2	0.97	0	0.00	3	3.80	10	3.13	1	1.64	7	2.06
**neoplastic**	364	45.50	225	44.91	126	47.91	176	44.22	187	46.75	42	30.00	321	48.78	50	36.50	43	38.39	109	52.91	17	48.57	28	35.44	148	46.39	14	22.95	173	51.03
**toxic**	10	1.25	5	1.00	5	1.90	5	1.26	5	1.25	4	2.86	6	0.91	2	1.46	2	1.79	1	0.49	0	0.00	2	2.53	3	0.94	2	3.28	3	0.88
**traumatic**	60	7.50	28	5.59	21	7.98	31	7.79	29	7.25	33	23.57	27	4.10	11	8.03	9	8.04	7	3.40	1	2.86	13	16.46	18	5.64	20	32.79	9	2.65
**unclassified**	735		493		214		371		358		132		597		157		129		169		29		68		303		64		294	

Regarding causes of death by organ system, the nervous system was the most commonly affected organ system causing death in all subcategories except in mixed breed and large dogs, which both had a slightly higher percentage of deaths from musculoskeletal disease. Causes of death by organ system are provided in Table 4.

**Table 4:** Causes of death classified by organ system in our data

Organ System	All Dogs	%	Purebreds	%	Mixed Breeds	%	Males	%	Females	%	Intact	%	Gonadectomized	%	Small	%	Medium	%	Large	%	Giant	%
**cardiovascular**	108	11.37	74	11.78	30	10.56	50	10.50	58	12.31	23	13.53	85	10.94	29	16.48	20	13.42	19	7.76	3	7.32
**dermatologic**	20	2.11	13	2.07	6	2.11	8	1.68	12	2.55	5	2.94	15	1.93	3	1.70	5	3.36	4	1.63	0	0.00
**endocrine**	28	2.95	20	3.18	7	2.46	15	3.15	13	2.76	3	1.76	25	3.22	7	3.98	5	3.36	6	2.45	0	0.00
**gastrointestinal**	90	9.47	64	10.19	22	7.75	48	10.08	42	8.92	21	12.35	69	8.88	14	7.95	15	10.07	28	11.43	5	12.20
**hematopoietic**	126	13.26	70	11.15	51	17.96	69	14.50	56	11.89	13	7.65	112	14.41	10	5.68	15	10.07	39	15.92	4	9.76
**hepatic**	57	6.00	40	6.37	13	4.58	31	6.51	26	5.52	5	2.94	52	6.69	16	9.09	8	5.37	13	5.31	0	0.00
**musculoskeletal**	142	14.95	86	13.69	54	19.01	66	13.87	74	15.71	25	14.71	115	14.80	8	4.55	12	8.05	55	22.45	11	26.83
**neurologic**	200	21.05	140	22.29	51	17.96	99	20.80	101	21.44	41	24.12	159	20.46	41	23.30	33	22.15	50	20.41	13	31.71
**ophthalmologic**	4	0.42	1	0.16	3	1.06	3	0.63	1	0.21	1	0.59	3	0.39	0	0.00	1	0.67	0	0.00	0	0.00
**respiratory**	92	9.68	59	9.39	26	9.15	48	10.08	44	9.34	18	10.59	74	9.52	23	13.07	16	10.74	17	6.94	3	7.32
**urogenital**	83	8.74	61	9.71	21	7.39	39	8.19	44	9.34	15	8.82	68	8.75	25	14.20	19	12.75	14	5.71	2	4.88
**unclassified**	585		366		193		293		287		102		478		118		92		130		23	

When separating dogs by both sex and gonadectomy status, we found that gonadectomized dogs of both sexes were more likely to die from diseases of the hematopoietic system and that this effect was more pronounced in females (*P* = 0.033, Fisher’s exact test). As for pathophysiologic processes, we found that gonadectomized dogs of both sexes were more likely to die from degenerative disease (*P* = 0.013, Fisher’s exact test) and neoplasia, with the latter effect being more pronounced in females (*P* = 0.0001, Fisher’s exact test). Sexually intact animals were more likely to die from infectious and/or inflammatory causes (*P* = 0.014, Fisher’s exact test) and were also more likely to die from trauma, this effect also being more pronounced in females (*P* = 1E-12, Fisher’s exact test).

## Discussion

In this report on the demographics and mortality of companion dogs seen at three independent US private veterinary practices in different regions, we identified important differences in MST and categorical causes of death from what has been reported previously for US dogs in more narrowly constructed samples. We demonstrated that electronic medical records data collection from private unaffiliated veterinary hospitals from varying settings and using diverse medical records software systems, and captured a description of the demographic distribution, lifespan and categorical causes of death of privately owned dogs in these clinics. A baseline understanding of such a diverse population will establish a demographic and lifespan description against which future studies of particular groups, specific risk factors, or health interventions can be compared. Based on the *One Health* and *One Welfare* paradigms [2, 51–53], data such as these may have implications for similar studies of human health.

As mentioned in the introduction, a limiting factor of many veterinary lifespan studies has been the fact that they were based on deceased animals only. While this is an appropriate method for studying causes of death, when using such data to study lifespan, this results in right censored data and artificially reduces measured lifespan [27, 28]. This study corrects for right censored data by considering both dogs known to have died and dogs known to have been alive at their most recent veterinary visits. Our measured one-year mortality of 7.3% is consistent with what one would expect to see in a diverse dog population [18].

We also found a strong inverse correlation between body size and lifespan, which reflects previous findings [18, 31]. Using Kaplan-Meier survival curves [29] and Cox proportional hazard models [30], we were then able to evaluate survival and death hazards within our patient population. Cox proportional hazard models in particular were useful to test the effects of multiple factors on observed lifespan separately, as we did in our analyses when correcting for body size, the strong effect of which may otherwise have interfered with most of our results. While measured weight ignores body condition that could change with age, we did not find any obvious differences in the percentage of dogs with incongruent grouping by age when comparing dogs whose size could be classified based on both measured weight and breed-specific weight (Supplemental Figure 1).

Using these methods, we did find a survival advantage in gonadectomized dogs over intact dogs of the same sex, which was stronger in females than in males. We also point out that any gonadectomized dog must by definition already have lived to the age at which the surgery was performed, which may represent another confounding factor leading to an overestimation of the lifespan advantage of gonadectomized dogs. In this context, it has also been suggested that the age of gonadectomy should be taken into account when analyzing veterinary epidemiological data [36]. While our data did not include information on age of gonadectomy, we attempted to correct for these issues by also testing the effects of gonadectomy on lifespan using only dogs that were known to have survived to at least age 5. In doing so, we found that the increased survival associated with female gonadectomy was conserved, while the effect found for male gonadectomy across all ages was not conserved in this group. All of this would support the view that gonadectomy is a sex-specific procedure with differential effects between males and females, and that gonadectomy should therefore be considered separately by sex in statistical analyses of veterinary epidemiology, as has been done in some previous studies [10].

Additionally, we also performed an analysis of the effects of female gonadectomy using only females that had lived to be at least 8 years old and found that the beneficial effects of gonadectomy were conserved in this population, indicating that the lack of a beneficial effect of gonadectomy on lifespan in female Rottweilers described previously [46] cannot be generalized to a more diverse dog population. Unfortunately, our data did not include a sufficient number of aged female Rottweilers to conduct a meaningful statistical analysis in this breed only.

Given that our data were sampled from a US-based population, where gonadectomy is widely considered to be part of responsible dog ownership, it is possible that in our dataset, being gonadectomized acts as a proxy for better husbandry and veterinary care for these dogs, which in turn might have influenced our results showing a higher life expectancy associated with gonadectomy. However, the large effect size in females is indicative that at least female gonadectomy does have a substantial beneficial effect on lifespan.

While it is likely that some of the dogs in our data were gonadectomized because of genital disease, this information was not available in the collected data. However, the relatively high prevalence of routine gonadectomy in the US dog population would indicate that any such effect would likely have been comparably small in relation to the effect of routine gonadectomy [54].

In comparing dogs by AKC functional groups and DNA-based ancestral groups, the most striking feature is the significantly lower lifespan in the Mountain ancestral group, which persists even when we correct for body weight and clinic, and the influence of which appears to be stronger than any AKC group-based differences. This is a novel finding that may indicate a genetic effect in this ancestral group accounting for a decrease in lifespan; however, it may also be that these dogs represent breeds that through their husbandry or through unrelated genetic effects are prone to shorter lifespans. While we did not have sufficient numbers of dogs to compare causes of death, it is worth pointing out that Bernese Mountain Dogs in particular are a member of this ancestral group and are known to be prone to early death due to a high incidence of histiocytic sarcoma [55, 56].

Our study did not find a significant survival advantage of mixed breeds and/or F1 hybrids over purebreds, in contrast with another recent study [45]. However, within purebred dogs, we saw evidence not only that lower overall inbreeding levels are associated with increased lifespan, consistent with [45], but also that lifespan was greater in breeds with higher effective population size as a whole, indicating that these two measures of genetic diversity are positively correlated with breed lifespan. It is worth mentioning that the mixed breed survival advantage has recently been shown to be more pronounced in large and giant breed dogs and that certain small and medium-sized purebreds live longer than mixed breeds of similar size [18], which may have influenced our findings in that more than half of all dogs in our population were small or medium-sized.

The overall MST for dogs in this study was 15.4 years which is longer than the mean lifespan reported by Greer et al. in an AKC breed-based study (12.2 yrs.) [31], longer than the mean age at death reported by Hoffman et al in a study of 20 years of VMDB data (9 yrs.) [10] and longer than median age at death reported by Patronek et al among mixed breed dogs in the VMDB (8.5 years) [57]. This is not surprising because both VMDB studies are reliant upon a population subject to referral bias [21], while the lifespan data in the AKC breed-based study was collected by voluntary reporting and recall. We also note that a recent study from a primary care environment that also corrected for right censored data found mean ages that are comparable to our present findings [18].

The systematic inclusion of data from all companion dogs seen by the three practices in this study over the period of a year reflects a more general population, and removes referral and recall bias. By contrast, a UK study using prospectively collected data from dogs attending 86 veterinary private practices reported a mean lifespan of 12.0 years [17]. The UK study design is similar to the study reported here and was not subject to recall or referral bias; however, it did not take right censored data into account, which may have contributed to the lifespan appearing to be shorter [27]. This difference may also reflect differences in the health or veterinary care of US vs UK dogs, or it is possible that the three US practices reported here are a biased group in some way and the mean lifespan derived from the larger number of UK practices is more generalizable. In addition, our data were somewhat skewed by the fact that Clinic B, from an urban environment, accounted for the majority (70%) of dogs in our data, while Clinics A and C together accounted for only 30%, which may have introduced bias towards a more urban environment. However, other than gonadectomy in males that had lived to at least 5 years of age, all other effects remained significant when we included clinic as a variable in the Cox models.

Differences in the most populous breeds within this study compared with prior studies may also have impacted the differences in estimates of lifespan. However, this factor is difficult to assess. Compared to the study by Hoffman et al. [10], only four of the most populous ten breeds (Labrador Retriever, Golden Retriever, German Shepherd Dog, Dachshund) were among the top ten breeds reported in this study; O’Neill et al. [17] also shared four of the top ten breeds with this study (Labrador Retriever, Golden Retriever, German Shepherd Dog, Yorkshire Terrier), but the order of popularity differed among the three studies, with the exception that the Labrador Retriever was the most populous breed in all three studies. The other six breeds in the top ten were unique to each study. The breed frequency in the study by Greer et al. [31] was not reported.

Similar studies have not all used the same system to classify causes of death, but where possible to compare, neurologic and musculoskeletal causes were the top two organ system causes of death reported in this study as well as in Hoffman et al. and O’Neill et al., and neoplasia was by far the leading pathophysiologic process cause of death in all three studies. Beyond those top listed causes, organ system and pathophysiologic process cause of death frequencies vary among the studies. Cause of death reporting is not a standard component of veterinary medical record-keeping, and imprecision as well as inter-rater variability in cause of death coding in all three studies is likely. However, it is also plausible that frequency of various causes of death does differ among US veterinary teaching hospitals, US private practices and UK private practices, and that may also exert an influence on the different lifespan estimates reported. Our data did not include information on whether a dog was insured; however, we would expect insured dogs to generally live longer than uninsured dogs of the same size due to the easier availability of expensive treatments where warranted.

While unlikely, it is possible that some dogs in the data may have attended more than one of the three clinics that were part of the present study. Given the different unique patient identifiers used in each clinic’s electronic patient records, we were unable to rule out this possibility. However, given the geographical distance between the three clinics as well as the fairly short duration of the study period, we believe that any such effect is unlikely to have significantly influenced our results.

## Conclusions

This study is the first US-based investigation of lifespan in dogs using data from independent private veterinary clinics. We show that such data can be obtained and standardized in practice to allow for meaningful analysis. Our findings confirm that lifespan inversely correlates with body size in dogs and identify differential associations with lifespan between male and female gonadectomy. Specifically, our results suggest that while gonadectomy is associated with increased lifespan in both males and females, we find a significantly greater effect in females. We also provide evidence that lower inbreeding and higher effective population size are associated with longer lifespans in purebred dogs, and that the Mountain ancestral group lives considerably less long than other purebred dog ancestral groups.

Future research is needed to investigate the importance of age at gonadectomy on lifespan in a sex-separated manner, as well as the factors that contribute to the shorter lifespan in the Mountain ancestral group. Furthermore, we argue that epidemiological data from veterinary practice such as these can also be correlated with data from human epidemiology from the same areas, which may prove useful in establishing the privately owned dog as a model organism for human epidemiology under a *One Health* paradigm [2].

## Methods

Medical records data were collected from three US veterinary clinics over a 12-month period from March 1st, 2014 to February 28th, 2015. As one objective of this study was to demonstrate the feasibility of large-scale electronic medical records abstraction from diverse, independent veterinary practices, clinics were chosen to reflect 1) urban, suburban and rural environments; 2) general, specialty and emergency care; and 3) three different proprietary electronic medical records systems. Clinic A is a small, rural, general and surgical specialty practice in Indiana with a caseload of 3400 dog visits per year. Clinic B is a large, urban, 24-h emergency, general and multispecialty hospital in Washington DC with a caseload of 27,000 dog visits per year. Clinic C is a medium-sized suburban general practice in Florida with a caseload of 6000 dog visits per year. The clinics use three different commercially available veterinary medical record software tools (AVImark by Henry Schlein, Melville, NY; Cornerstone by IDEXX, Westbrook, ME; and ImproMed by Henry Schlein, Melville, NY). One of the investigators (KEC) traveled to each clinic to devise a system for data extraction from each software tool that suited that clinic’s daily workflow and required minimal new work effort on the part of clinic staff. Data were reported monthly for all patients seen and included a unique patient identifier, date of birth, date of visit, sex, gonadectomy status, body weight, and owner-reported breed. If any patient of the practice died at the clinic or was reported dead by the owner, the data also included date of death and categorical cause of death assignments for organ system (OS) and pathophysiologic process (PP), as determined by the attending veterinarian. Because post-mortem examinations are not routinely performed in veterinary practice, the option of “unclassified” was available for both OS and PP categories [9]. Client-identifying information was not collected. Each monthly report was manually standardized upon receipt by one of the the authors (KEC). Incomplete entries were identified and individual records were reviewed by a representative at each clinic to complete entries when possible.

Given that multiple entries of the same dog would have led to statistical issues regarding survival and mortality estimates by artificially increasing the percentage of live dogs in the data, multiple demographic entries for the same dog over the course of the studied year were identified based on unique patient identifier, and only the most recent visit date was analyzed for each individual patient. If the patient was recorded as being alive at that date, the most recent visit date was considered the point of censoring.

Data were analyzed using R [58]. Package survival was used to create graphs of survival curves and perform statistical tests related to survival analysis. Kaplan-Meier curves were compared using Log Rank tests; multivariate testing (including correction for body size and clinic) was conducted using Cox Proportional Hazard models through the coxph function in package survival. Comparisons of causes of death by organ system and pathological process were conducted using Fisher’s exact test. A *P* value of less than or equal to 0.05 was considered statistically significant.

Dogs were classified into four size classes defined as small (under 20 lbs), medium (20–40 lbs), large (40–90 lbs), and giant (over 90 lbs), as used in [33]. This classification was carried out using both measured weight at most recent visit, and weight according to American Kennel Club (AKC) breed standard averages if the dog was classified as a purebred. When using measured weights, dogs under 18 months of age were excluded from weight-related analyses to avoid bias due to juvenile large and giant dogs; however, when using AKC breed standard average weights, purebred dogs under 18 months of age were included based on projected adult weight for their breed.

For the purposes of analysis, dogs were classified as purebreds, mixed breeds, or hybrids in the data according to the patient chart, with “hybrid” denoting F1 crosses between purebreds. Purebreds were further categorized based on the seven American Kennel Club (AKC) breed groups (Herding, Hound, Non-sporting, Sporting, Terrier, Toy and Working), as well as the five ancestral breed groups defined by Parker et al. (2012) [47] (Ancient, Herding-Sighthound, Mastiff-Terrier, Modern and Mountain).

Considering the important physiological and anatomical differences between the sexes, we tested the lifespan effects of gonadectomy separately by sex. Given that the observed survival advantage in gonadectomized dogs may be at least in part caused by the fact that any gonadectomized dog needs to already have lived to the age at which it underwent the surgery [36], we then repeated these analyses using only dogs that were at least 5 years old, which should eliminate most of this bias. Because it has been proposed that being intact may provide a survival advantage in female Rottweilers aged at least 8 years [46], we also tested whether the survival advantage of gonadectomized females remained significant when only analyzing dogs known to have lived to be at least 8 years old.

There is some concern regarding possible negative health effects of inbreeding in purebred dogs [59]. Based on this, we tested for an effect of inbreeding and effective population size in the purebred dogs in our data. Average inbreeding and effective population size measures by breed were taken from the literature [48, 50] and purebred dogs were classified into a below-median and an above-median category for each of these measures according to their breed to create survival curves. Both average inbreeding and effective population size were also analyzed as continuous variables specific to each breed in separate Cox models.

## Supplementary information

**Additional file 1: Supplemental Table 1.** List of F1 hybrid dogs in the data and their percentage in relation to all F1 hybrids. **Supplemental Table 2.** Comparison of sex, gonadectomy status, measured body size, and median survival time (including 95% confidence interval) by clinic and overall.

**Additional file 2: Supplemental Figure 1.** Comparison between purebred dogs aged at least 18 months by weight class as determined by measured or breed standard-based weight. There is no obvious correlation between the percentage of inconsistently classified dogs and age.

## Data Availability

The data used for this study are available from the corresponding author on reasonable request.
