# Supplementary material for: Lifespan of companion dogs seen in three independent primary care veterinary clinics in the United States
Source: Canine Med Genet. 2020 Jun 16;7:7. doi: 10.1186/s40575-020-00086-8 (PMC7386164; doi:10.1186/s40575-020-00086-8)
Supplement: Supplementary file 1 — Additional file 1: Supplemental Table 1. List of F1 hybrid dogs in the data and their percentage in relation to all F1 hybrids. Supplemental Table 2. Comparison of sex, gonadectomy status, measured body size, and median survival time (including 95% confidence interval) by clinic and overall. [file 40575_2020_86_MOESM1_ESM.docx]

| **F1 Hybrid** | **Parent Breeds** | **N** | **%** |
| --- | --- | --- | --- |
| Aussiedoodle | Australian Shepherd X Poodle | 3 | 0.3% |
| Bernedoodle | Bernese Mountain Dog X Poodle | 1 | 0.1% |
| Cavachon | Cavalier King Charles Spaniel X Bichon Frisé | 42 | 4.1% |
| Cavapoo | Cavalier King Charles Spaniel X Poodle | 23 | 2.3% |
| Cockapoo | American Cocker Spaniel X Poodle | 228 | 22.4% |
| Goldendoodle | Golden Retriever X Poodle | 263 | 25.9% |
| Labradoodle | Labrador Retriever X Poodle | 248 | 24.4% |
| Maltipoo | Maltese X Poodle | 51 | 5.0% |
| Peekapoo | Pekingese X Poodle | 25 | 2.5% |
| Pomapoo | Pomeranian X Poodle | 3 | 0.3% |
| Puggle | Pug X Beagle | 68 | 6.7% |
| Schnoodle | Schnauzer X Poodle | 60 | 5.9% |
| Terripoo | Terrier X Poodle | 1 | 0.1% |

**Supplemental Table 1**: List of F1 hybrid dogs in the data and their percentage in relation to all F1 hybrids

|  | **Clinic A** | **Clinic B** | **Clinic C** | **Total** |
| --- | --- | --- | --- | --- |
| **Total Males** | 1,268 | 7,654 | 1,760 | 10,682 |
| **Intact Males** | 462 (36.4%) | 1,270 (16.6%) | 383 (21.8%) | 2,115 (19.8%) |
| **Gonadectomized Males** | 806 (63.6%) | 6,384 (83.4%) | 1,377 (78.2%) | 8,567 (80.2%) |
| **Total Females** | 1,403 | 6,997 | 1,862 | 10,262 |
| **Intact Females** | 450 (32.1%) | 757 (10.8%) | 344 (18.5%) | 1,551 (15.1%) |
| **Gonadectomized Females** | 953 (67.9%) | 6,240 (89.2%) | 1,518 (81.5%) | 8,711 (84.9%) |
| **Small** | 961 (35.9%) | 4,227 (29.3%) | 1,123 (31.7%) | 6,311 (30.6%) |
| **Medium** | 711 (26.6%) | 4,824 (33.4%) | 791 (22.3%) | 6,326 (30.6%) |
| **Large** | 753 (28.2%) | 4,679 (32.4%) | 1,422 (40.1%) | 6,845 (33.2%) |
| **Giant** | 249 (9.3%) | 703 (4.9%) | 207 (5.8%) | 1,159 (5.6%) |
| **Median Survival Time (Years)** | 15.7 (15.0-16.5) | 15.6 (15.3-15.9) | 14.9 (14.5-15.2) | 15.4 (15.2-15.7) |

**Supplemental Table 2**: Comparison of sex, gonadectomy status, measured body size, and median survival time (including 95% confidence interval) by clinic and overall.
