# Supplementary material for: Lifespan of companion dogs seen in three independent primary care veterinary clinics in the United States
Source: Canine Med Genet. 2020 Jun 16;7:7. doi: 10.1186/s40575-020-00086-8 (PMC7386164; doi:10.1186/s40575-020-00086-8)
Supplement: Supplementary file 2 — Additional file 2: Supplemental Figure 1. Comparison between purebred dogs aged at least 18 months by weight class as determined by measured or breed standard-based weight. There is no obvious correlation between the percentage of inconsistently classified dogs and age. [file 40575_2020_86_MOESM2_ESM.docx]

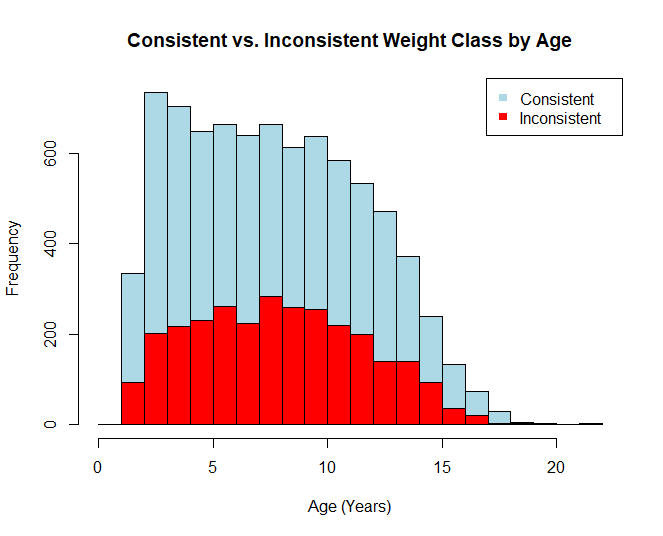


**Supplemental Figure 1**: Comparison between purebred dogs aged at least 18 months by weight class as determined by measured or breed standard-based weight. There is no obvious correlation between the percentage of inconsistently classified dogs and age.
